# Improvement of Multiparametric MR Image Segmentation by Augmenting the Data With Generative Adversarial Networks for Glioma Patients

**DOI:** 10.3389/fncom.2020.495075

**Published:** 2021-01-27

**Authors:** Eric Nathan Carver, Zhenzhen Dai, Evan Liang, James Snyder, Ning Wen

**Affiliations:** ^1^Henry Ford Health System, Detroit, MI, United States; ^2^Wayne State University, Detroit, MI, United States

**Keywords:** GaN, U-net, glioma, GBM, segmentation

## Abstract

Every year thousands of patients are diagnosed with a glioma, a type of malignant brain tumor. MRI plays an essential role in the diagnosis and treatment assessment of these patients. Neural networks show great potential to aid physicians in the medical image analysis. This study investigated the creation of synthetic brain T1-weighted (T1), post-contrast T1-weighted (T1CE), T2-weighted (T2), and T2 Fluid Attenuated Inversion Recovery (Flair) MR images. These synthetic MR (synMR) images were assessed quantitatively with four metrics. The synMR images were also assessed qualitatively by an authoring physician with notions that synMR possessed realism in its portrayal of structural boundaries but struggled to accurately depict tumor heterogeneity. Additionally, this study investigated the synMR images created by generative adversarial network (GAN) to overcome the lack of annotated medical image data in training U-Nets to segment enhancing tumor, whole tumor, and tumor core regions on gliomas. Multiple two-dimensional (2D) U-Nets were trained with original BraTS data and differing subsets of the synMR images. Dice similarity coefficient (DSC) was used as the loss function during training as well a quantitative metric. Additionally, Hausdorff Distance 95% CI (HD) was used to judge the quality of the contours created by these U-Nets. The model performance was improved in both DSC and HD when incorporating synMR in the training set. In summary, this study showed the ability to generate high quality Flair, T2, T1, and T1CE synMR images using GAN. Using synMR images showed encouraging results to improve the U-Net segmentation performance and shows potential to address the scarcity of annotated medical images.

## Introduction

Approximately 121,000 (Ostrom et al., 2018) people in the US are diagnosed with a malignant brain tumor annually, with over 13,000 of those being Glioblastoma (GBM), defined by the World Health Organization (WHO) as grade IV tumors with an unacceptable median overall survival despite best available treatment of less than to 2 years. For primary brain tumors WHO grade II-IV, there are no curative treatments and limited approved therapies. Current management of primary brain tumors has two standard benchmarks, tissue analysis for diagnosis, and the longitudinal analysis of treatment response/ tumor stability through serial brain tumor imaging. In fact, the brain MRI in patients with GBM is used to stratify clinical trial options prior to initial surgery and to offer patients definitive cytoreduction surgery for malignant glioma or GBM when radiographic features are highly suggestive of a malignant tumor. Therefore, advanced imaging methods to stratify patients into phenotypic, functional, molecular, and prognostic groups is highly sought after.

Amongst GBM researchers, clinicians, patients, and patient advocates there is hope that new advances as promised by molecular targeted therapies, advanced radiation techniques, evolving surgical technologies, and unforeseen innovation will result in improved patient outcomes. One key element to all of these are MR images; both for diagnosis and longitudinal patient monitoring. Applications of deep machine learning in brain tumor imaging has the potential to transition from a subjective analysis to objective analysis and create a new set of tools to refine treatment options, improve care quality, and ultimately impact patient care. One critical limitation to achieving the success seen in non-medical imaging is the volume of data needed to power deep machine learning. It is common knowledge that deep learning techniques are highly powerful when there are numerous training samples. However, in the medical field, especially in clinical trials, where limited numbers of training samples are accessible, deep learning models are easily overfitting during the training stage and perform poorly in prediction (Shen et al., 2017). Besides, annotation of medical images is generally expensive, time-consuming, and requires highly trained clinicians. Therefore, data argument has been widely used to increase the original dataset to improve the performance of supervised learning. One possible solution to overcoming the limited brain tumor imaging data available for analysis is to create synthetic brain tumor MR images. Synthetic MR (synMR) images of sufficient quality may be created using a generative adversarial network (GAN). Herein, we quantitively and qualitatively evaluated the quality of these created synMR and established the capability of using synMR images for the practical application of increasing the volume of data required by deep learning. Specifically, we evaluated the performance of image segmentation using a widely implemented two-dimensional (2D) U-Net model (Ronneberger et al., 2015) by augmenting real patients' T1-weighted (T1), post-contrast T1-weighted (T1CE), T2-weighted (T2), and T2 Fluid Attenuated Inversion Recovery (Flair) MR images data with varying amount of synMR images. In fact, the investigation of the changes in accuracy of enhancing tumor (ET), whole tumor (WT), and tumor core (TC), also known as the non-enhancing necrotic region, for glioma patients when incorporating varying amounts of synMR images may be the most practically useful metric in judging both quality and real-world usability of T1, T1CE, T2, and Flair synMR.

## Method

### Patient Population

Data was obtained from the BraTS multimodal Brain Tumor Segmentation Challenge 2018 (Menze et al., 2015; Bakas et al., 2017a,b, 2018). Nineteen different institutions provided a total of 210 patients for training and 66 patients for validation. T1, T1CE, T2, and Flair MR images were provided for each patient. Provided ET/WT/TC contouring was performed by one to four clinicians and approved by neuro-oncologists.

### Image Pre-processing

BraTS provided T1, T2, and Flair MRI that were rigidly registered with T1CE, resampled (1 × 1 × 1 mm^3^) and skull stripped. In addition to the pre-processing performed by BraTS, this study performed normalization and padding of each 2D MRI slice from 240 × 240 to 256 × 256. To aid in data balance between tumor and unlabeled areas, the z dimension in the training dataset was cropped to 64 slices from original 155 slices. This served to decrease amount of unlabeled data present during training and increase focus on the tumor regions for data augmentation and segmentation. Data augmentation was done by flipping each slice left/right to decrease dependence on location as the brain exhibits marked symmetry across the sagittal plane. No cropping was performed on the validation dataset as all 155 slices were segmented during validation.

### Generative Adversarial Neural Network

We developed an augmentation network to create synMR images as a new augmentation approach to aid in overcoming the well-known limitation of available annotated medical image data. We manipulated semantic label maps of lesions in real MR (rMR) images, e.g., changing lesion locations or types, and then transferred the new label to synMR image using the augmentation network. Compared to traditional augmentation methods such as affine transformation or cropping, which could not guarantee standard anatomical structures, this approach introduced new data augmentations by varying tumor sizes, shapes and locations while maintained the authentic morphologic structures of brain.

#### Architecture

**Figure 1:**
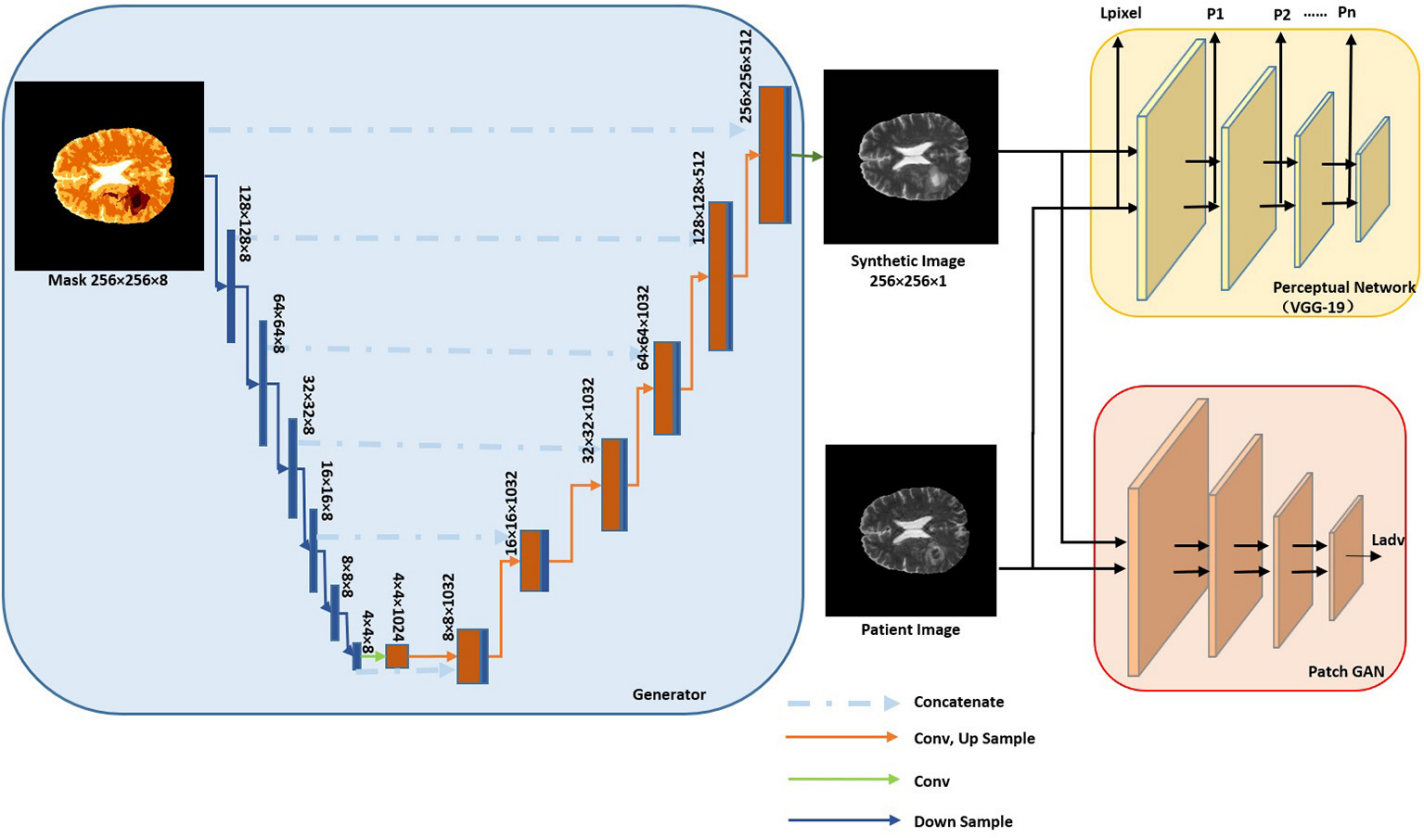
Architecture of Augmentation Net. The Generator is shown in blue box and discriminators in yellow and red boxes.

Our augmentation network consisted of a generator (blue box in Figure 1) and two discriminators (red and yellow box in Figure 1). The generator was used to generate synMR images from sematic label maps which in turn were derived from rMR images. The semantic label map was composed of normal brain tissue and GBM tumor segments. GBM segments were further classified into the ET/WT/TC regions derived from T2 rMR. Three quarters, one half and a quarter of maximum pixel values of T2 were the three categorizing thresholds that were used to segment normal brain tissues. Five categories of segments were generated in total. The discriminators were used to distinguish between synMR images and rMR images.

#### Generator

The generator consists of several components *Ci*-„ with each operating at a different resolution. The semantic label (*256* × *256*) is down sampled to provide segmentation layout at the different resolutions (*wi* × *hi, wi* = *hi*). The first component, *C0*, gets down-sampled semantic labels at the resolution of *w0* = *h0* = *4* as input and then it generates feature maps as an output for the next component. For components *C1* to *Cn*, feature maps from previous component are up sampled at a scale of 2 and are concatenated with the semantic labels of the same resolution as input. A residual block is applied to generate feature maps as output. The convolution kernel size is *3*×*3*, layer normalization (Gomez-Iturriaga et al., 2016) is applied, and ReLU (Maas et al., 2013) is used as the activation function.

#### Discriminator

Two discriminators are used. The first one is a pre-trained VGG-19 convolutional neural network (Simonyan and Zisserman, 2014), which won the first and second place in localization and classification in the Image Net Large Scale Visual Recognition Challenge (ILSVRC) 2014. It is used to calculate the perceptual loss ($\sum_{i}p_{i}$) and the image per-pixel loss $L_{im}$.

(1) $$\begin{array}{l} L_{im}=\;\underset{m}{\sum }\underset{n}{\sum }|I_{real}-I_{synthetic}| \end{array}$$

*I*_*real*_ represents the real patient rMR image, *I*_*synthetic*_ represents the synMR image created by the generator, with $\underset{m}{\sum }\underset{n}{\sum }\;$ indicating the summation over all pixels

(2) $$\begin{array}{l} p_{i}=\;\underset{m}{\sum }\underset{n}{\sum }|{\theta_{i}}_{real}-{\theta_{i}}_{synthetic}| \end{array}$$

*p*_*i*_is the perceptual loss from layer *i* of VGG-19 Net. Perceptual loss was firstly proposed by Johnson et al. (2016) and was claimed to be more robust than image per-pixel loss to measure image similarities. θ_*ireal*_ and θ_*isynthetic*_ are feature maps of rMR image and synMR images generated at layer *i*, respectively. The second one is a patch GAN, which penalizes on image patches, the loss is given as $L_{adv}=\;𝔼\left[D\left(I_{real},I_{synthetic}\right)\right]+𝔼\left[1-D\left(I_{synthetic}\right)\right]$. *D*(.) is the discriminator net. The total loss is computed as the weighted summation of each loss.

The synMR image is generated by solving the following objective:

(3) $$\begin{array}{l} S^{*}=argmin\left(𝔼\left[\overset{n}{\underset{i=0}{\sum }}\lambda_{i}p_{i},+\lambda_{im}L_{im}\right]+\lambda L_{adv}\right) \end{array}$$

#### Training and Generation of New Training Samples

One hundred sixty-four patients were randomly selected from the BraTS18 dataset for training. For each MRI modality, an independent model was trained. λ_*i*_, λ_*im*_ and λ were adapted every 10 epochs to maintain the balance among each loss. The total training epoch was 100 for each modality. New semantic labels were created from real labels to augment synMR images. The lesion contours were rotated with a random angle (0°-90°), translated with a random number (0–40) of pixels and randomly flipped left/right/up/down. The lesion contours that were outside the brain contour were changed to zero (background). Then the augmented semantic labels were used as the new training dataset and transferred into image domain using the augmentation network of each imaging modality.

#### GAN Evaluation Metrics

Mean Square Error (MSE), Mean Absolute Error (MAE), Peak Signal to Noise Ratio (PSNR), and Structural Similarity Index (SSIM) were used to quantitatively compare between the synMR and rMR images.

In MSE (Equation 4) variable “n” represents number of images being compared. Since MSE depends on intensity scaling, it is necessary to report these details. In this study, 16-bit images were used with the pixel range 0–255.

(4) $$\begin{array}{l} MSE=1/\text{n }\sum \left\{{\left(original\;image-generated\;image\right)}^{2}\right\} \end{array}$$

MAE (Equation 5) determines the prediction error between the rMR and synMR.

(5) $$\begin{array}{l} MAE=1/\text{n }\sum \left\{\left(original\;image-generated\;image\right)\right\} \end{array}$$

PSNR (Equation 6) overcomes the limitation of MSE by scaling the MSE value according to image range, which is done by the S^2^ value in Equation 4. Generally, the higher the PSNR, the better the synthetic image; however, this metric has a limitation.

(6) $$\begin{array}{l} PSNR\;\left(dB\right)=-10*log_{10}\left(\frac{MSE}{S^{2}}\right) \end{array}$$

SSIM shows the perceived change in structural information as opposed to MSE, MAE, and PSNR that show absolute error differences. SSIM assumes pixels close to each other possess strong inter-dependency. It is based on luminance, contrast, and structure differences between the images and is among the most commonly used metrics to compare the synthetic images to the original images.

The benchmark of quantitative metrics of our study deviated from other works on synthetic images as one of the key characteristics of our methodology was to produce variations of tumor size, shape and location in synMR. Therefore, there were inherent differences between the synMR and rMR which made direct quantitative comparisons difficult. To overcome this limitation, qualitative analysis of synMR images was performed in the form of the Turing test, physician individual synMR review, and investigation of changes in deep learning performance. The Turing test requires a physician to correctly classify a dataset consisting of both rMR and synMR. A misclassification percentage of fifty percent implies the rMR and synMR are indistinguishable. In addition to this test, an in-depth analysis of a randomly selected synMR was performed by an authoring physician. SynMR images were also assessed for the practical application of increasing the volume of data required by deep learning. Specifically, synMR was incorporated both in subgroups and as a whole during training of the outlined U-Net segmentation model. Investigation of impact on performance of segmentation could provide feedback on the quality of synMR images.

### U-NET Segmentation Model

The segmentation model is comprised of three individual 2D U-Nets designed by Ronneberger et al. (2015), one for each of the three tumor regions: ET/WT/TC. Each U-Nets was trained with rMR and synMR images of modalities T1, T1CE, T2, and Flair. This model combines the ET/WT/ET contours generated by the three separate U-Nets during post-processing. Two processing techniques were used to improve the segmentation model's ability to accurately contour ET/TC. The first one served to aid the segmentation model during training by mathematically manipulating input T1 MRI to improve delineation of ET/TC boundaries. Specifically, each input T1 MRI was used in conjunction with its corresponding T1CE MRI and the pixel-wise intensity difference between these MRI was calculated. This calculated array replaced the T1 MRI during training. The second technique was to use the WT contour as a boundary for ET/TC delineations. Therefore, any ET/TC contour predicted outside of the WT contour would be erased. The best model for each type of contour was chosen according to the validation loss within 100 epochs run on GPU (Titan XP, nVidia, Santa Clara, CA).

#### U-NET Architecture

Each U-Net followed Pelt and Sethian (2018) recommendation of four downscaling and upscaling layers. Each downscaling layer is followed by a batch normalization layer (Pelt and Sethian, 2018) and the architecture uses this grouping to downsize the image while increasing the number of features. Each upscaling layer is merged with its corresponding downscale layer and used to return the downsized image to the size of the original. These layers combined to form a merged layer and soft dice (Equation 7) was employed as the loss function.

(7) $$\begin{array}{l} Dice\;Loss=\frac{2*<y_{true,}y_{pred}>+c}{<y_{true,}y_{true}>+<y_{pred,}y_{pred}>+c} \end{array}$$

*y*-_true_ is the clinician's contour, *y*_*pred*_ is the model's output, and *c* (0.01) is a constant to avoid division-by-zero singularities.

#### Creation of Training Datasets

As outlined previously, synMR images were generated from 210 GBM patients' rMR. To further investigate how synMR could affect segmentation performance during training of the U-Net, the synMR images were randomly partitioned into four unique subsets. Multiple U-Nets were trained using the total rMR in combination with each of these synMR subsets. One U-Net was trained using only rMR to serve as a baseline with which to compare performance. Four other U-Nets were trained on datasets that contained either a quarter, half, three-quarters or total generated synMR to investigate how the amount of synMR incorporated in the training dataset influences model performance. In order to solely evaluate the impact of amount of synMR images on the model performance, extra care was taken to decrease variance of the quality of synMR used in each training datasets. This was accomplished by dividing all synMR into four subsets equally, with each subset containing an exclusive quarter of all available synMR. These subsets were then numerically labeled one through four and used in the following manner to create the training datasets. Subset one was used to form the training dataset containing one quarter of synMR. To form the training dataset that employed half of the generated synMR, subset one was combined with subset two. Similarly, subsets one, two, and three were used to form training dataset representing three-quarters of available synMR, while all four subsets were used for the total synMR dataset. By staggering synMR subsets in each training model, we could evaluate the model performance differences with regards to change in the amount of synMR incorporated.

#### U-NET Evaluation Metrics

Dice similarity coefficient (DSC), Hausdorff distance with 95% confidence interval (HD), sensitivity, and specificity are used to evaluate U-Net segmentation as these metrics quantitatively show the agreement between the created U-Net model and the “gold-standard” physician created contours. Specifically, DSC indicates volumetric agreement of the physician created contour and the contours generated in this study. Reported DSC values fall into the range zero to one with zero indicating no volumetric overlap and one indicating complete volumetric agreement. HD indicates point-based agreement between the compared contours. This quantitative metric shows largest relevant Euclidean offset between every pixel in the ground truth contour and its corresponding pixel in the generated contour.

(8) $$\begin{array}{l} h\left(A,B\right)=max_{a\in A}\left\{min_{b\in B}\left\{d\left(a,b\right)\right\}\right\} \end{array}$$

with *a* and *b* being points of sets *A* and *B*, respectively, and *d(a,b)* is the Euclidean metric between these points (Menze et al., 2015).

Sensitivity (true positive) and specificity (true negative) indicate level of border agreement between generated and physician contours. While DSC shows volumetric overlap of contours, these metrics report relative size differences. Essentially, they report if the generated contour is smaller or larger than the physician's contour.

(9) $$\begin{array}{l} Sensitivity=\\ \frac{number\;of\;true\;positives}{number\;of\;true\;positives\;+\;number\;of\;false\;negatives} \end{array}$$

(10) $$\begin{array}{l} Specificity=\\ \frac{number\;of\;true\;negatives}{number\;of\;true\;negatives\;+\;number\;of\;false\;postives} \end{array}$$

## Results

### SynMR Quantitative Analysis

MSE, MAE, PSNR, and SSIM were performed to provide quantitative analysis of synMR. Table 1 shows quantitative metric for each modality. Consistent inter-modality results demonstrate that high similarity is achieved between the rMR and synMR for all modalities (T1,T1CE,T2, Flair).

**Table 1:** Average reported Mean Square Error (MSE), Mean Absolute Error (MAE), Peak Signal to Noise Ratio (PSNR), and Structural Similarity Index (SSIM) for synMR images generated by GAN.

	**MSE**	**MAE**	**PSNR**	**SSIM**
T1	19.3 ± 0.3	23.4 ± 0.6	43.1 ± 0.4	0.788 ± 0.002
T1CE	19.2 ± 0.3	22.8 ± 0.6	43.1 ± 0.4	0.789 ± 0.004
T2	19.2 ± 0.3	23.4 ± 0.4	43.1 ± 0.5	0.784 ± 0.003
Flair	18.9 ± 0.4	24.1 ± 1.5	43.1 ± 0.5	0.794 ± 0.005

### SynMR Qualitative Analysis

Qualitative analysis was performed to further investigate both overall and inter-modality synMR quality. Specifically, qualitative analysis of synMR was assessed in two ways by an authoring physician. The first assessment was the performance of the Turing test (Table 2). The second one was an in-depth visual comparison of generated synMR images with their corresponding rMR images on each of the following modalities: T2, Flair, T1, and T1CE.

**Table 2:** The classification accuracy of a subset of synMR and original images reviewed by the physician blindly.

**Modality**	**% Misclassified**
Flair	26.3
T1	10.5
T1CE	26.3
T2	26.3

*The amount of synMR and rMR improperly categorized by physician is represented by percent misclassified*.

#### SynMR Qualitative Analysis: Turing Test

A subset of 9 rMR images and 10 synMR images flair, T1, T1CE, and T2 MR images were randomly selected for evaluation. The physician was presented with each of these 19 MR images blindly and judged if the MR image was rMR or synMR and provided feedback. Ideally the rMR and synMR images would be completely indistinguishable from each other, and this would be reflected by a 50 percent misclassification rate of the images. As shown in Table 2, Flair, T1CE, and T2 MR images were misclassified 26.3 percent of the time, while T1 was incorrectly identified 10.5 percent of the time. This lower score was due to the visible streaking artifacts on coronal and sagittal views for some of the synMR images.

#### SynMR Qualitative Analysis: In-Depth Physician Analysis

**Figure 2:**
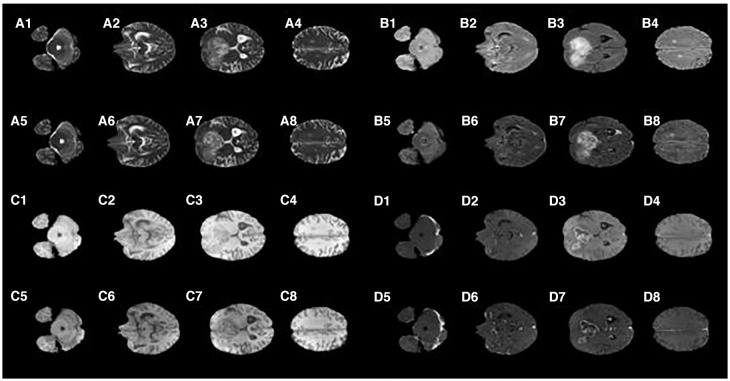
Synthetic MRI compared with patient MR images. **(A1–A4)**, synthetic T2; **(A5–A8)**, patient T2; **(B1–B4)**, synthetic Flair; **(B5–B8)**, patient Flair; **(C1–C4)**, synthetic T1; **(C5–C8)**, patient T1; **(D1–D4)**, synthetic T1CE; **(D5–D8)**, patient T1CE.

Figure 2 shows that the T2 MR image's main difference between the synMR and rMR lay in the tumor at the right frontal lobe (lower left on the images A3 vs. A7). It was noted that the tumor geometry was preserved, but the relative signal intensities in the region were distorted. Specifically, synMR differed in appearance in the core of the tumor, as it displayed a hyperintense T2 signal compared with the surrounding edema. In addition, the signal from edema was also slightly different in images A4 and A8. Image A8 had a broader range of contrasts within the edema, whereas A4 delineated the extent of the edema with a sharper drop-off at the edges than the rMR. Comparing the edema between Flair rMR images and synMR images (B3 vs. B7), it showed differences in the extent of the edema. Also, there were noted circumferential artifacts in the rMR flair images (B1–B4). T1 MR images showed that quality of synMR (C1–C4) was very good. For T1CE MR images, the boundary of enhanced rim and necrotic regions of the T1CE synMR (D1–D4) were clearly defined, although the area surrounding the tumor had slight decrease in intensity.

In summary, synMR had high image quality with clearly defined structural boundaries. However, synMR suffered in showing details inside lesions and areas of high gradient (e.g., edema signal in T2 modalities). It was possible that this detailed information was lost when lesion pixels were classified into the same semantic label. This is notable as re-gaining lost information is a well-known GAN limitation.

### U-Net: Utilization of SynMR

In addition to quantitative and qualitative investigation of synMR image quality, incorporation of generated synMR in training datasets for the U-Net segmentation model was done to assess the ability of synMR to enhance segmentation performance. The impact on the U-Net's segmentation performance by incorporating synMR during training is indicative of both quality and synMR's capabilities as a data distillation technique. SynMR was evaluated both as a whole set and in overlapping subsets containing either a quarter, half, or three quarters of the synMR images.

#### U-Net: DSC/HD Analysis

**Figure 3:**
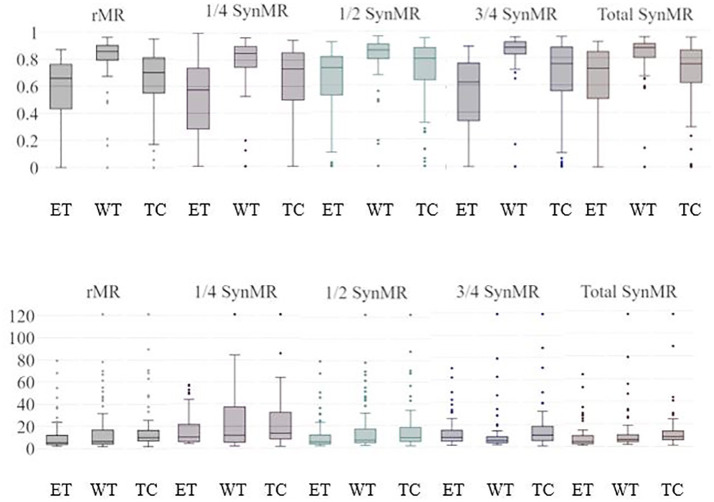
ET/WT/TC Validation Results for U-Nets trained by BraTS MRI and different subsets of synMR(None, 1/4,1/2,3/4, total synMR incorporated). Top row shows DSC, bottom shows HD. Each grouping displays results in order ET/WT/TC. Incorporation of synMR above threshold ratio of 2:1 (rMR:synMR) improves DSC and HD. Standard box-plot format used with singular points displaying outliers.

The two most popular metrics, DSC and HD, are used to identify ET, WT, and TC segmentation performance for the U-Nets. Figure 3 shows the DSC and HD for each structure of the validation dataset trained with different subsets of synMR.

Figure 3 shows standard box-plot results for both DSC and HD for each model. *T*-Test reported statistically significance in models containing one quarter, half, and total synMR when compared against baseline. In addition, the relationships between neighboring models as synMR increased showed statistical significance as well. It can be seen that U-Nets trained using at least half synMR show a direct relationship between the amount synMR used and the U-Net performance. The statistical significance in model relationships combined with differences in model performance (Figure 3) indicate that a threshold ratio of 2:1 (rMR:synMR) is necessary to introduce more variance while maintaining a proper distribution of data. HD shows significant improvement; however, DSC shows lower relative improvement as DSC is inherently biased in this study due to the fact that it was used as the loss function during the training of each U-Net.

#### U-Net: Sensitivity/Specificity Analysis

While sensitivity and specificity are not as integral in judging the quality of generated contour as DSC/HD, they show the level of accuracy in defining the tumor border, as well as the size differences between the ground truth and generated contours. As the U-Net was trained, DSC was optimized, however, this metric only indicates the level of volumetric overlap, which leaves the size of the generated contour dependent on other factors. These factors can relate to the differences in training datasets and give insight into how incorporation of synMR changes the U-Nets. Table 3 shows that when one half or a quarter of synMR was implemented, sensitivity and specificity both increased, indicating improvement in border definition. However, when all synMR was used, sensitivity decreased while specificity remained relatively unchanged. Since synMR showed higher distinction from rMR at the boundaries with sharper gradient drop off, this could lead to a systematic difference of the segmentation labels between the two datasets and led to smaller contours generated from U-Net. However, the smaller contour generated by training on synMR possessing gentler gradients does not negatively affect overall U-Net performance, as specificity and sensitivity mainly show the direction of the offset between the ground truth and generated contours (HD).

**Table 3:** Validation results for U-Nets trained by BraTS MRI and different subsets of synMR.

**Percent of SynMR added**		**None (rMR Only)**	**1/4**	**1/2**	**3/4**	**All**
Sens.	ET	0.70	**0.80**	**0.84**	**0.78**	0.62
	WT	0.89	**0.90**	0.89	0.83	0.80
	TC	0.66	**0.74**	**0.75**	**0.77**	0.66
Spec.	ET	0.96	**0.99**	**0.99**	**0.99**	**0.99**
	WT	**0.99**	0.98	**0.99**	**0.99**	**0.99**
	TC	**0.99**	**0.99**	**0.99**	**0.99**	**0.99**

*Bolded values indicate highest contour metric value*.

### Individual Cases

**Figure 4:**
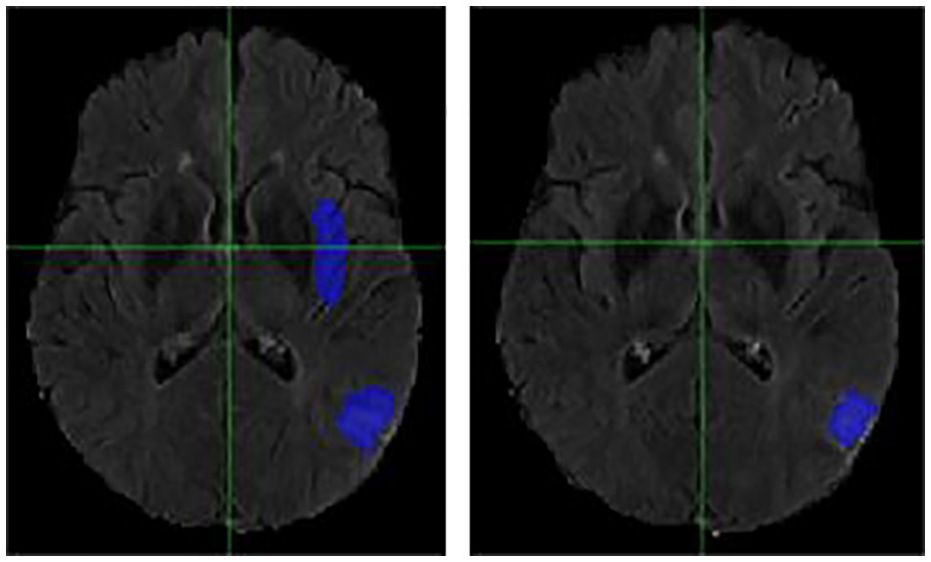
Case One (Improved). Flair MRI. rMR only **(Left)** and Total SynMR MRI **(Right)** DSC of WT was improved from 0.21 to 0.67.

**Figure 5:**
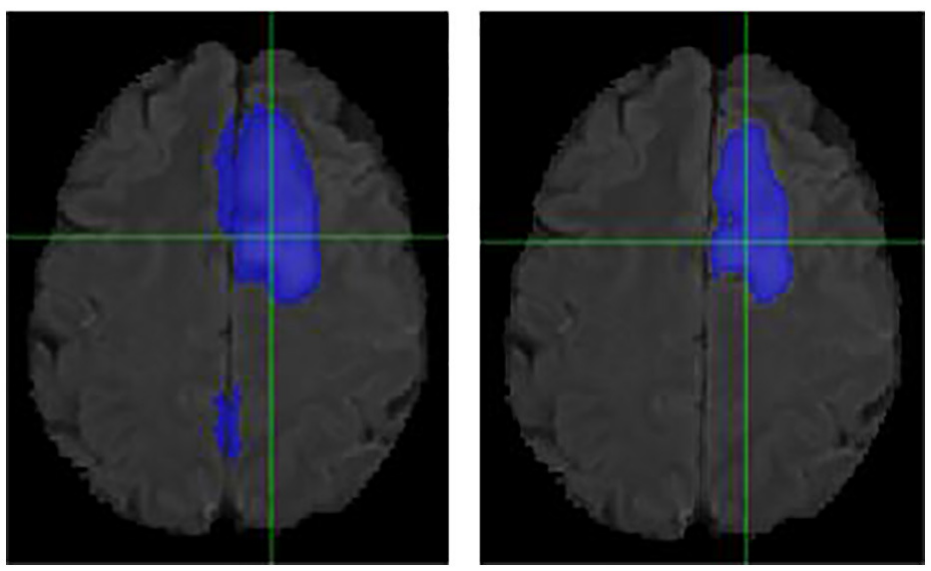
Case Two (Improved). Low-grade Glioma. Flair MRI. rMR only **(Left)** and Total SynMR MRI **(Right)** DSC of WT was improved from 0.49 to 0.88.

**Figure 6:**
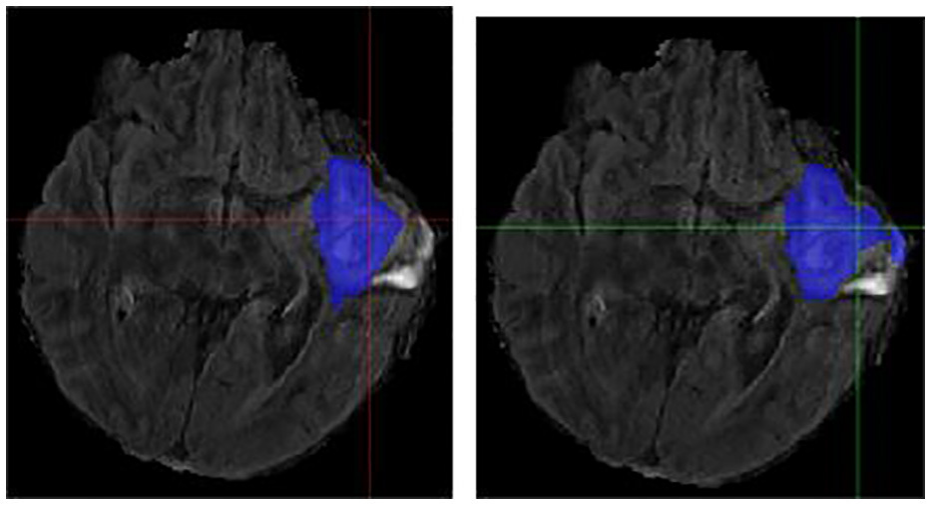
Case One (Worsened). Flair MRI. rMR only **(Left)** and Total SynMR MRI **(Right)** DSC of WT changed from 0.76 to 0.58.

**Figure 7:**
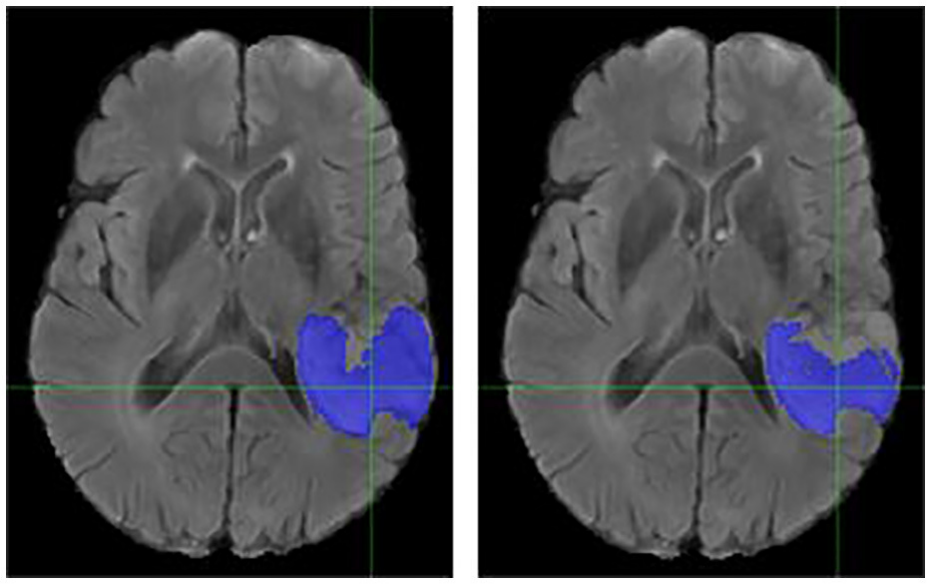
Case Two (Worsened). Low-Grade Glioma. Flair MRI. rMR only **(Left)** and Total SynMR MRI **(Right)** DSC of WT showed a decrease from 0.86 to 0.59.

It is necessary to outline the best and worst cases to assess the model performance. The best performing and worst performing individual cases are carefully evaluated. Figures 4–7 show the comparison of contours on WT for two good and two poor performing cases.

We have observed encouraging improvement of the segmentation accuracy for high grade glioma when the lesion was centrally and radially located. However, the challenge still exists in the low-grade glioma cases due to increased difficulty in boundary definition. Location also plays a role in discerning whether the contouring accuracy would improve or not. The improved low-grade glioma case was centrally located, while in the case that did not show improvement was located toward the edge of the brain. It can also be seen in the improved cases (Figures 4, 5) that the U-Net focuses more on differences in structure, rather than differences in pixel intensity. This is in line with the strength of synMR, as synMR quality regarding structure outperforms its quality pertaining to intensities.

## Discussion

The original idea of synthesizing images indistinguishable from reality is inspired by the development of GANs (Goodfellow, 2014). GANs have been employed to expand training datasets for many tasks. Specifically, synthesizing new images as training samples provides a possible solution to overcome the challenge of the limited number of annotated medical images. Frid-Adar et al. (2018) achieved impressive results in lesion classification using GAN-synthesized images, which indicated the potential of GAN for data distillation tasks. Bowles et al. (2018) used GAN for segmentation, however, there are important differences between the studies. First, they experimented on image patches sampled from the dataset, while we experimented on the entire images (Bowles, 2018). Second, their study generated synMR images and contours from Gaussian noise (Bowles, 2018). Due to this, their study was not able to provide a quantitative evaluation between rMR and synMR images. Their work was limited to only providing a visual comparison using the Turing test (section SynMR Qualitative Analysis).

Researchers have leveraged GANs in a conditional setting which allows the model to deterministically control the generation of particular samples based on external information (Gauthier, 2014; Mirza, 2014; Isola, 2017) However, some researchers suggested that adversarial training might be unstable or even diverge, and introduced image per-pixel loss and perceptual loss (Dosovitskiy and Brox, 2016) that was used in this study. In this paper, we proposed an augmentation network that was trained in a supervised fashion using paired semantic labels and rMR images. This method was chosen over (Frid-Adar, 2018) and (Bowles, 2018) as it incorporated flexible object manipulations with desired scenarios, which allows for the creation of synMR with various tumor size and location from those present in the rMR images.

Our study explored the idea to utilize GAN to generate synthetic images to improve image segmentation performance. Specifically, the segmentation model employed the previously outlined 2D U-Net (Ronneberger et al., 2015) due to its effectiveness, competitiveness and high familiarity, as it has been widely adopted in the field of image segmentation. In the medical field, especially in clinical trials, where limited numbers of training samples are accessible, deep learning models can easily overfit during training and perform poorly in application on independent datasets (Ronneberger et al., 2015). Purely increasing the size of the training dataset by simple inclusion of synMR does not guarantee higher performance. However, the neural networks performance will show improvement if the synMR is of sufficient quality and introduce diversity. This study assumes that the model performance is sufficient to judge the overall data distillation ability of synMR generated in this study. We postulate that that all neural network-based segmentation models should show improvement if trained on datasets containing more variance, although level of improvement may vary model to model. However, this study recognizes that this should be further investigated in a future study by introduction of one or more additional segmentation neural networks.

“TumorGan” (Qingyun Li et al., 2020) and “ANT-GAN” (Sun et al., 2020) were different GAN methodologies to generate synMR. Direct quantitative comparison of synMR image quality is difficult among studies due to synMR/rMR structural differences. Specifically, the tumor location in our synMR was purposefully changed from original rMR to increase variability of resulting datasets. This structural difference between synMR and rMR created the need for advanced qualitative analysis by authoring physicians. However, compared to the other two studies, our work showed competitive results on the improvement of segmentation using synMR as a data augmentation technique. The other two studies reported an increase over baseline of 2.6 and 2.5% in the average DSC while our study showed an improvement of 4.8%.

Statistical investigation of incorporation of certain subsets of synMR during training showed improved performance when incorporating synMR at or above the threshold ratio of 2:1 (rMR to synMR). This ratio could be due to the inherent necessity to introduce additional variance in the training dataset. However, differences in individual synMR image quality could also play a role. Even though the subsets of synMR were staggered in the training models, there are still differences in the quality of individual synMR images. This difference in individual synMR qualities could play a part in the reasoning behind reduced results in segmentation performance when trained using only one randomized subset of synMR. The difference in individual synMR qualities can be partially explained by the fact that rMR quality was not constant. Individual rMR quality differed as it was obtained from different MRI machines over many years. Since image quality had been improved throughout this time, recently obtained rMR images generally show a higher image quality than older rMR. Performance of the employed GAN was impacted by this as it is not likely that the generated synMR will possess greater quality than its corresponding input rMR. However, the relationship of individual synMR quality and its impact regarding the model's performance should be further investigated.

## Conclusion

We were able to generate high quality Flair, T2, T1, and T1CE synMR using the presented augmentation network and had a thorough evaluation of the images both quantitatively and qualitatively. In addition, the synMR images proved their capability as a data augmentation technique, as incorporation of the created synMR images to increase the size and diversity of the training dataset showed promising results. The presented data manipulation strategy has the potential to address the challenges regarding the limited labeled medical dataset availability for medical image segmentation.

## Data Availability Statement

All datasets generated for this study are included in the article/Supplementary Material.

## Author Contributions

NW designed the research and methodology. EC and ZD performed the data pre-processing, machine learning training, and analysis. EC, NW, ZD, and JS wrote the paper. EL and JS provided clinical guidance on the evaluation of image quality and segmentation accuracy.

## Conflict of Interest

The authors declare that the research was conducted in the absence of any commercial or financial relationships that could be construed as a potential conflict of interest.
